# Exploring Genomic Variations in Nematode-Resistant Mutant Rice Lines

**DOI:** 10.3389/fpls.2022.823372

**Published:** 2022-03-24

**Authors:** Manoranjan Dash, Vishal Singh Somvanshi, Jeffrey Godwin, Roli Budhwar, Rohini Sreevathsa, Uma Rao

**Affiliations:** ^1^Division of Nematology, ICAR-Indian Agricultural Research Institute, New Delhi, India; ^2^Bionivid Technology Private Limited, Bangalore, India; ^3^ICAR-National Institute for Plant Biotechnology, New Delhi, India

**Keywords:** rice, *Meloidogyne graminicola*, resistance, mutants, SNPs, InDels, genetic variations, genome

## Abstract

Rice (*Oryza sativa*) production is seriously affected by the root-knot nematode *Meloidogyne graminicola*, which has emerged as a menace in upland and irrigated rice cultivation systems. Previously, activation tagging in rice was utilized to identify candidate gene(s) conferring resistance against *M. graminicola*. T-DNA insertional mutants were developed in a rice landrace (acc. JBT 36/14), and four mutant lines showed nematode resistance. Whole-genome sequencing of JBT 36/14 was done along with the four nematode resistance mutant lines to identify the structural genetic variations that might be contributing to *M. graminicola* resistance. Sequencing on Illumina NovaSeq 6000 platform identified 482,234 genetic variations in JBT 36/14 including 448,989 SNPs and 33,245 InDels compared to reference indica genome. In addition, 293,238–553,648 unique SNPs and 32,395–65,572 unique InDels were found in the four mutant lines compared to their JBT 36/14 background, of which 93,224 SNPs and 8,170 InDels were common between all the mutant lines. Functional annotation of genes containing these structural variations showed that the majority of them were involved in metabolism and growth. Trait analysis revealed that most of these genes were involved in morphological traits, physiological traits and stress resistance. Additionally, several families of transcription factors, such as FAR1, bHLH, and NAC, and putative susceptibility (S) genes, showed the presence of SNPs and InDels. Our results indicate that subject to further genetic validations, these structural genetic variations may be involved in conferring nematode resistance to the rice mutant lines.

## Introduction

Rice (*Oryza sativa* L.) is the staple food crop for ~3.5 billion people and is one of the essential cereal crops for human nutrition and food security. Global rice demand is estimated to reach 852 million tons by 2035 (Khush, 2013). However, several abiotic and biotic stresses constrain rice production, including a broad range of pathogens and pests. The rice root-knot nematode (RRKN) *Meloidogyne graminicola* Golden and Birchfield, 1965 is an important pest of rice and is reported to cause up to 80% yield loss (Mantelin et al., 2017), whereas other nematode parasites of rice cause a 10%–25% yield loss (Luc et al., 2005; Kyndt et al., 2014). *Meloidogyne graminicola* is reported to be the most damaging plant-parasitic nematode (PPN) in India. Out of the total loss of USD 1.58 billion caused by various PPNs in India, RRKN alone was responsible for ~ 23% loss (*ca*. USD 313 million; Kumar et al., 2020). Resistant cultivars play a significant role in the management of RRKN in the absence of ample nematicides and other nematode management strategies (Mantelin et al., 2017). Several RRKN-resistant germplasms have been identified previously (Plowright et al., 1999; Cabasan et al., 2018; Galeng-Lawilao et al., 2018; Zhan et al., 2018; Hatzade et al., 2020). In most instances, the natural resistance needs to be transferred to agronomically desirable backgrounds/cultivars, which is time-consuming. Insertional mutagenesis with either T-DNA or transposons is a new and quick way to generate novel traits in high-yielding cultivars (An et al., 2005). In a previous study, a forward genetic screen for resistance to RRKN in an indica rice landrace JBT 36/14 genetic background identified four activation tagged mutants line-8, line-9, line-11, and line-15 (Hatzade et al., 2019a). These mutant lines showed post-penetration resistance to RRKN and reduced nematode multiplication factor as compared to the wild-type JBT 36/14 and a popular basmati rice cultivar Pusa Basmati 1121 (Hatzade et al., 2019a,b).

The availability of rice genome sequence and advancements in next-generation sequencing (NGS) technologies have enabled rapid identification of the genomic and genetic diversity of various rice germplasm and facilitated their utilization for the genetic enhancement of rice. Genetic polymorphisms cause phenotypic variations in traits in response to environmental stimuli. These variations have been the basis for the development of several molecular markers used in genetic analysis, e.g., restriction fragment length polymorphism (RFLP) and simple sequence repeats SSR (Jones et al., 2009). The detection of sequence polymorphisms, such as single-nucleotide polymorphisms (SNPs) and insertions/deletions (InDels), is one of the most important advantages of NGS technologies (Varshney et al., 2009; Huang et al., 2013). SNPs are being employed in breeding programmes for marker-assisted and genomic selection, association and QTL mapping, positional cloning, haplotype and pedigree analysis, seed purity analysis, and variety identification (McCouch et al., 2010). In addition, InDels have also been successfully used for marker-assisted selection, fine mapping, QTL mapping, varietal testing (Hayashi et al., 2006; Steele et al., 2008; Vasemägi et al., 2010; Liang et al., 2011) and have the potential for map-based cloning of genes (Pan et al., 2008).

The position of SNPs and InDels within a genome can affect both gene expression and function (Shastry, 2009; Haraksingh and Snyder, 2013; Lin et al., 2017). DNA polymorphisms present within coding regions are critical in this context as they might alter the function of a protein. In addition, genetic variations present in regulatory sequences are also significant, as they can induce/repress gene expression, thus modulating gene function. Therefore, the discovery of polymorphisms related to phenotypes is important for understanding gene functions. In the present study, we performed whole-genome resequencing of four RRKN resistant mutant rice lines (lines 8, 9, 11, and 15) and JBT 36/14 landrace to identify genome-wide structural genetic variations and their possible role in conferring resistance against RRKN.

## Materials and Methods

### Plant Materials, DNA Isolation, and Genome Sequencing

The seeds of rice landrace JBT 36/14 were obtained from NRRI, Cuttack, India. The seeds from activation tagged mutants developed from JBT 36/14 background were provided by Dr. Rohini Sreevathsa, ICAR-NIPB, New Delhi. The seeds were germinated, leaf tissue was collected from 21-day old plants, and genomic DNA was isolated using the CTAB method. Qubit 2.0 fluorometer (Thermo Fisher) was used to quantify and NanoDrop 2000 (Thermo Fisher) to assess the quality of the isolated DNA.

For Illumina sequencing, the library was prepared by The NEBNext® Ultra™ II FS DNA Library Prep Kit as per manufacturer’s specifications. The quantity and size distribution of the libraries was estimated by Bioanalyzer 2100 (Agilent Technologies). The quantified libraries were subjected to whole-genome sequencing on the Illumina NovaSeq 6000 platform (Illumina Technologies) by paired-end sequencing to generate 150-base pair long reads. Standard Illumina pipeline was used to filter the whole genome sequencing data. To remove low-quality reads and reads containing adaptor/primer contamination, FASTQ files were further subjected to stringent quality control using NGSQC Toolkit v2.3 (Patel and Jain, 2012). Stringent criteria of 70:30 was used to obtain high-quality filtered reads wherein more than 70% HQ bases, each having Phred scores >30, were considered further for downstream analysis.

### Bioinformatic Analyses

**Figure 1:**
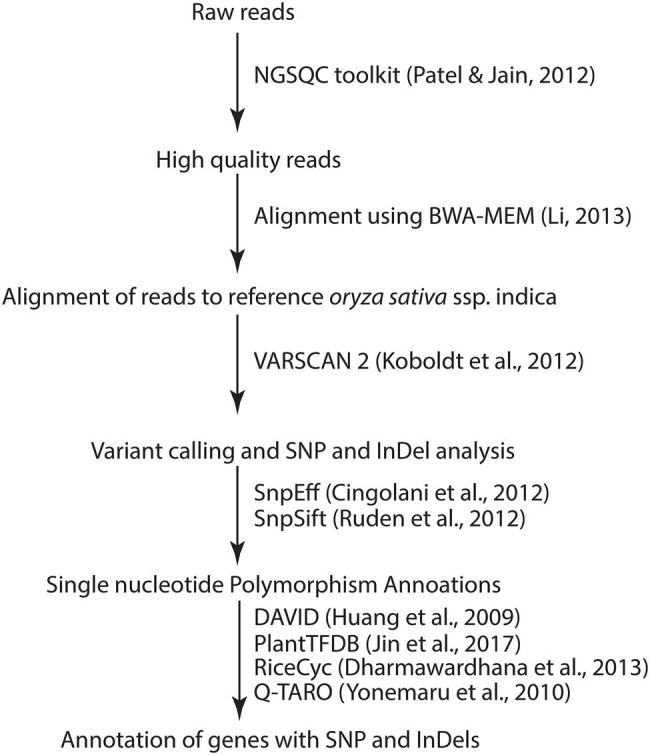
Pipeline followed for the analysis of NGS data in this study.

The general flow of bioinformatic analyses is presented in Figure 1. The high-quality filtered reads were mapped against *Oryza sativa* ssp. indica reference genome assembly (GCA_000004655) downloaded from Ensembl Plants (Howe et al., 2020) using BWA-MEM v1 (Li, 2013). The alignments were stored in BAM files. Duplicate read alignments were removed using SAMtools v0.1.16 (Li et al., 2009). Variants in the form of SNPs and small InDels were called using VarScan 2 (Koboldt et al., 2012). SNP annotations were done through SNPEff v5 (Cingolani et al., 2012) and SNPSift (Ruden et al., 2012). Circos was used to visualize the distribution of the SNPs and InDels on rice chromosomes (Krzywinski et al., 2009). All pathways associated with different variations were annotated using rice metabolic pathway database (RiceCyc; Dharmawardhana et al., 2013) and KEGG using default parameters. Gene ontology analysis was performed through DAVID (Huang et al., 2009). QTLs/Genes morphological, physiological and resistance/tolerance traits were downloaded from the Q-TARO database (Yonemaru et al., 2010). *O. sativa* ssp. indica transcription factors were downloaded from PlantTFDB (Jin et al., 2017). Previously characterized S genes (van Schie and Takken, 2014) of *Arabidopsis* and *O. sativa* were taken, and comparative sequence analysis was done with *O. sativa* ssp. indica genes. Variants related to these putative S genes were identified at the common genomic location in all mutant lines compared to JBT 36/14.

## Results

### Whole-Genome Sequencing of JBT 36/14

Whole-genome shotgun sequencing of JBT 36/14 genomic DNA on Illumina NovaSeq 6,000 platform yielded 81.57 million paired-end reads of 151 bp, containing 92.54% HQ reads (= reads > Phred quality score Q30; Table 1). These reads provided 30X coverage of the JBT genome. The data have been deposited in the NCBI SRA database (accession number PRJNA721239, SRX10576230). Further, the reads were aligned to the reference *Oryza sativa* ssp. indica 93-11 genome assembly GCA_000004655 and 98.3% of the total reads could be mapped to the reference genome with an average depth of 29 (Table 2).

**Table 1:** Raw data statistics of the sequenced rice lines.

Raw					Filtered		
Sample No.	Sample name	Sample description	Total no. of reads (million)	Total no. of bases (Gb)	Total HQ reads (reads ≥70% HQ bases)	%HQ Reads	% HQ bases^*^ in HQ reads
1.	JBT 36/14-1	Nematode Susceptible Rice Land Race Forward reads	40.78	6.15	37.74	92.54	93.95
2.	JBT 36/14-2	Nematode Susceptible Rice Land Race Reverse reads	40.78	6.15	37.74	92.54	91.05
3.	Line-8-1	Nematode resistant mutant Forward read	45.06	6.80	41.93	93.04	93.56
4.	Line-8-2	Nematode resistant mutant Reverse Read	45.06	6.80	41.93	93.04	91.59
5.	Line-9-1	Nematode resistant mutant Forward read	53.67	8.10	50.07	93.29	94.07
6.	Line-9-2	Nematode resistant mutant Reverse Read	53.67	8.10	50.07	93.29	91.42
7.	Line-11-1	Nematode resistant mutant Forward read	47.57	7.18	44.05	92.59	93.92
8.	Line-11-2	Nematode resistant mutant Reverse Read	47.57	7.18	44.05	92.59	91.06
9.	Line-15-1	Nematode resistant mutant Forward read	41.77	6.30	39.18	93.80	94.53
10.	Line-15-2	Nematode resistant mutant Reverse Read	41.77	6.30	39.18	93.80	91.59

* *HQ Bases > 30 Phred score*.

**Table 2:** Alignment of HQ reads on reference genome and average sequencing depth of each line in this study.

Genotype name	Alignment %	Average sequencing depth
JBT 36/14	98.3	29
Line-8	98.3	32
Line-9	98.2	38
Line-11	98	33
Line-15	98	30

**Figure 2:**
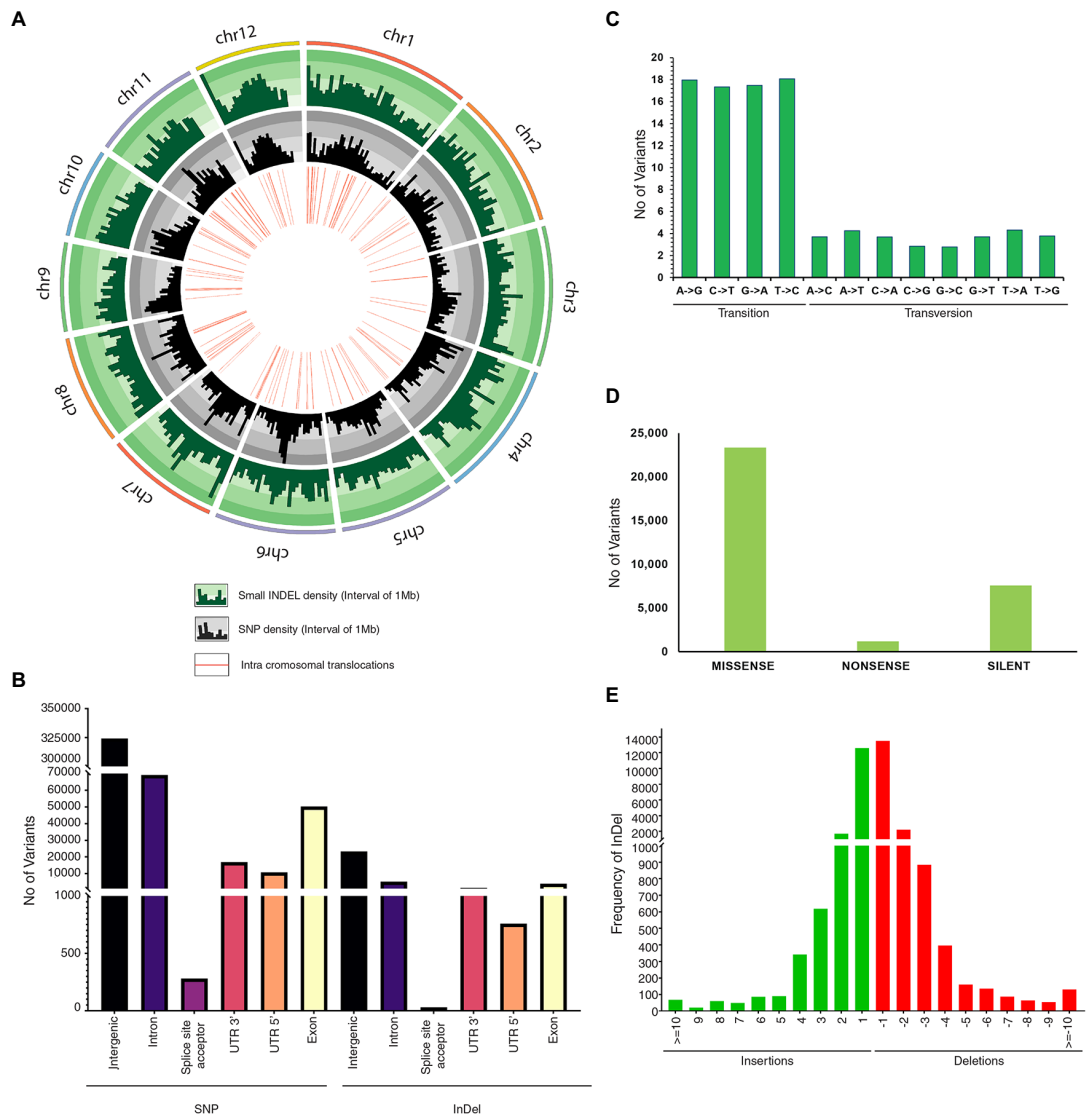
Genetic variation in JBT 36/14 compared to reference indica genome; **(A)** Circos diagram of genome wide variations depicting InDel density, SNP density and intrachromosomal translocations; **(B)** Annotation of SNPs and InDels by location; **(C)** Type of SNP variants by base substitution in JBT 36/14 genome; **(D)** Functional annotation of SNP variants; and **(E)** Size distribution of identified InDels in JBT 36/14 genome.

Comprehensive genome-wide densities of SNPs, insertions, deletions, and intra-chromosomal translocations in the JBT 36/14 genome are represented in Figure 2A. A total of 482,234 variations (448,989 SNPs and 33,245 InDels) were identified in JBT 36/14 genome as compared to the reference genome (Table 3; Supplementary File 1). The chromosome wise distribution of SNPs revealed maximum density of variations at the centromere region of chromosomes (Figure 2A). The majority of SNP variations (66.07%) were heterozygous, whereas most of the InDels were homozygous (60.18%; Table 3). For comparing nucleotide substitutions, all SNPs were subdivided into transitions (Ts) and transversions (Tv). Most of the SNP changes observed were of transition type with a Ti/Tv ratio of 2.40. Among transitions, T → C variations (18.07%) were most prominent, whereas T → A variations (4.32%) were the most frequent variation for transversions (Figure 2C). The 33,245 InDels ranged in size from 1 to 48 bp for deletions and 1 to 38 bp for insertions. A majority (90.23%) of the identified changes were short InDels of length 1–2 bp (Figure 2E). Of the total variations, 39,744 (8.24%) mapped to the coding region of the genome (Table 3). Chromosome (Chr) 1 shows the highest SNP and InDel density, while the lowest SNP density and InDel density were observed in Chr 9 (Supplementary File 1).

**Table 3:** Summary statistics of various identified structural variations in JBT 36/14 compared to reference genome *Oryza sativa* ssp. indica 93-11.

SNPs	
Total SNPs	448,989
Coding region variants	36,777
Homozygous variants	152,347
Heterozygous variants	296,652
Ti/Tv ratio	2.409
**InDels**	
Total InDels	33,245
Coding region variants	3,967
Homozygous variants	20,007
Heterozygous variants	13,238

The regions in which all polymorphisms were located in JBT 36/14 are summarized in Figure 2B. In JBT 36/14 genome, the majority of the SNP variations span the intergenic region (72%), whereas, among genic variations, the majority are intron variations (~15%) followed by exon variations (~11%) and 3’ UTR variations (~3%). A similar trend was found among the InDel variations, with the majority being in the intergenic region (~69%; Figure 2B). Among the total SNPs, 14.9% of variations pre-existed in reference indica genome 93–11 (from ensembl plant database), whereas 85.1% variations were novel as identified through variant effect predictor tool.^1^ Of the total variations in coding regions, 23,358 SNPs were missense variations and 7,572 synonymous variations with 1,185 variations being nonsense (stop gained) SNPs (Figure 2D).

### Whole-Genome Sequences of JBT 36/14 Mutants

**Figure 3:**
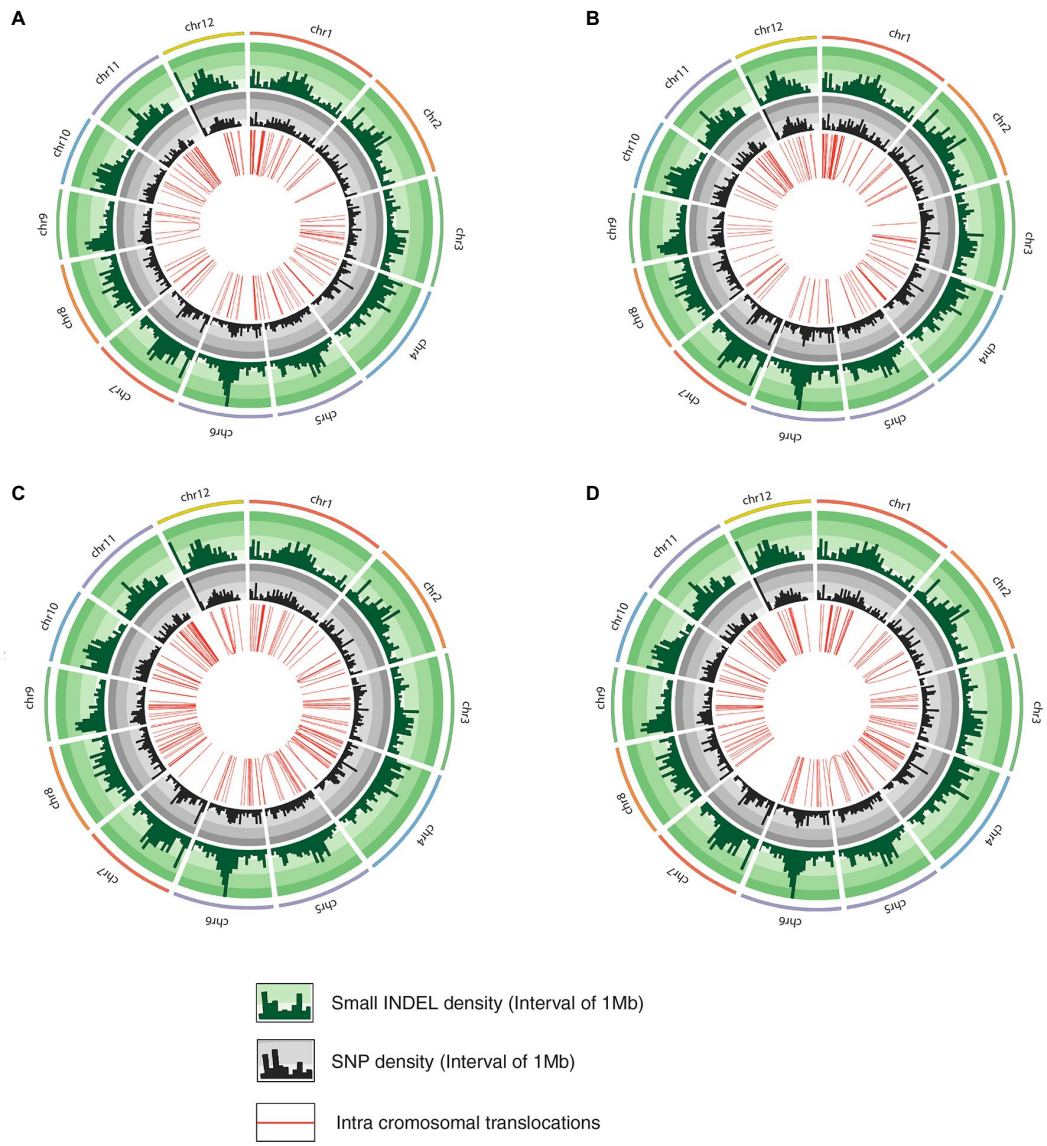
Circos diagram representing genetic variation in mutant rice lines compared to JBT 36/14 genome depicting InDel density, SNP density and intrachromosomal translocations; **(A)** line-8, **(B)** line-9; **(C)** line-11, and **(D)** line-15.

**Figure 4:**
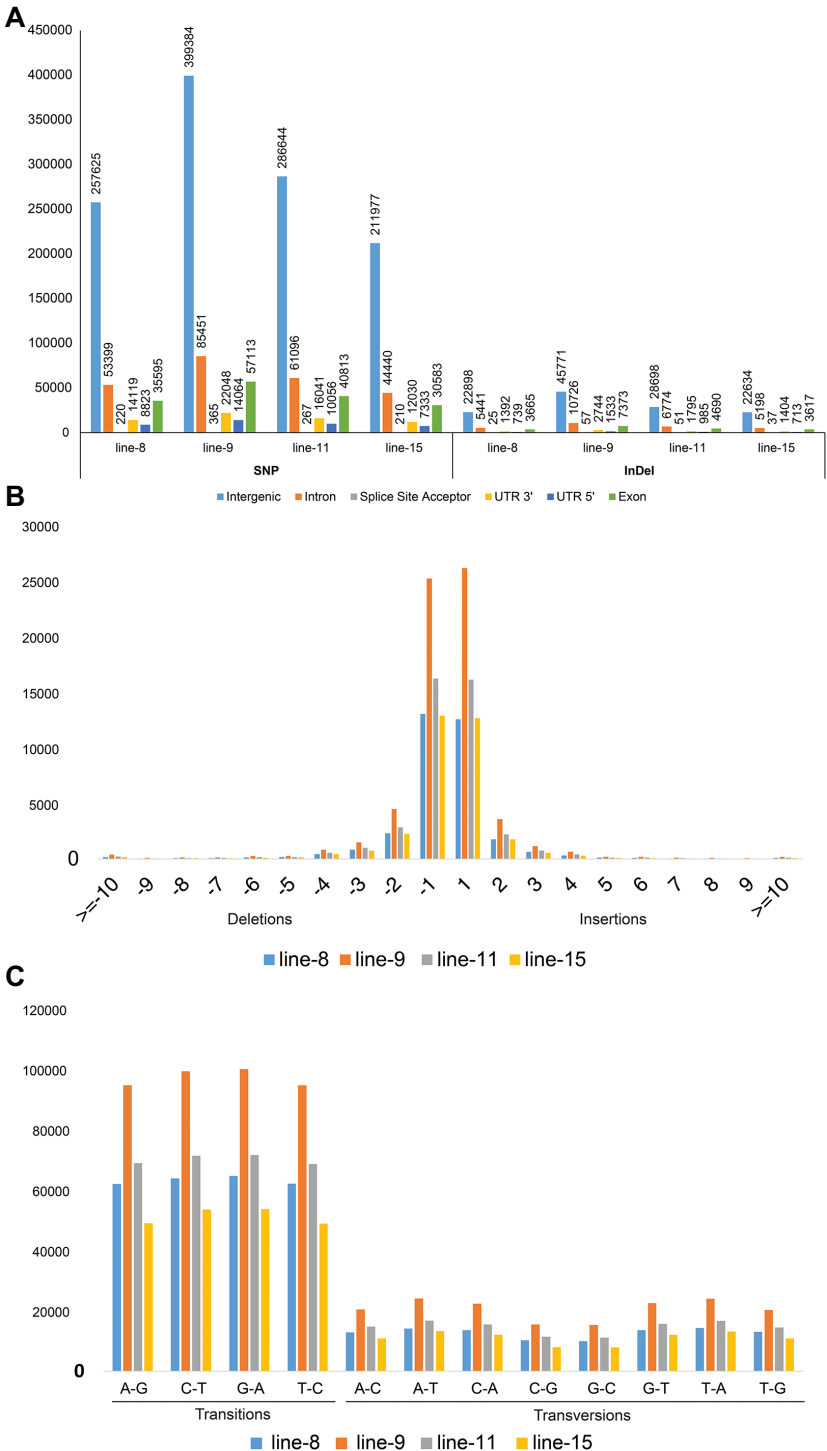
Annotation of SNP and InDel variants identified in mutant lines 8, 9, 11, and 15; **(A)** Annotations of SNP and InDel variants by their location; **(B)** Size distribution of identified InDels; and **(C)** Type of SNP variants by base substitution in mutant lines.

The whole-genome shotgun sequencing of mutant lines (line-8, line-9, line-11, and line-15) yielded 83.54–107.34 million paired reads of 151 bp for each sample (Table 1). The raw reads have been deposited in the NCBI SRA database (BioProject accession number PRJNA721239, SRX10576231—SRX10576234). More than 92.54% of the raw reads for each sample exceeded Phred quality score Q30 and were considered HQ reads. More than 98% HQ reads aligned to the reference *Oryza sativa* ssp. indica genome with an average depth of >29X (Table 2). Comprehensive genome-wide mapping of SNP, insertion, deletion and intrachromosomal translocations in the lines 8, 9, 11 and 15 genomes as compared to JBT 36/14 are presented in Figures 3A–D. As compared to JBT 36/14 genome, 354,340, 553,463, 396,978 and 293,237 SNPs were identified in line 8, 9, 11, and 15, respectively. Further, 963, 1,037, 960, and 907 SNPs were unique to lines 8, 9, 11, and 15 compared to JBT 36/14 and reference indica genome. Additionally, 32,885, 65,572, 41,296, and 32,395 InDels were identified in line 8, 9, 11, and 15, respectively, and 100, 162, 115, and 102 InDels were unique as compared to JBT 36/14 as well as the reference indica genome (Table 4). The majority of SNP (>52%) and InDel (>75%) variants were homozygous in the mutants. Like JBT 36/14, most of the SNP variants observed were of transition type with a Ti/Tv ratio ranging from 2.30 to 2.45 in the lines (Table 4). Transitions, G → A (~18%) and C → T (~18%) were found more frequently than A → G (~17%) and T → A (~17%) in all lines (Figure 4C). For transversions, A → T (~4%) was the most frequent, followed by T → A (~4%), C → A (~4%) and A → C (~4%), which were found at similar frequencies in all lines, while G → C (~3%) was least frequent in all lines (Figure 4C). The identified InDels ranged from 1 to 45 bp in size for deletions and 1–34 bp for insertions. The majority (~90.23%) of the identified variations were short InDels of length 1–2 bp (Figure 4B). Of the total variations, 56,681–90,023 SNPs mapped to the coding region of the genome. In all lines, Chr 1 showed the highest SNP and InDel density, while the lowest SNP density and InDel density were observed in Chr 9 (Supplementary File 1).

**Table 4:** Summary of unique structural variations in mutant rice lines as compared to reference genome *Oryza sativa* ssp. indica 93-11 and JBT 36/14.

Mutants	Line-8	Line-9	Line-11	Line-15
**SNP variants**				
Unique SNPs compared to JBT 36/14	354,340	553,463	396,978	293,237
Unique SNPs compared to JBT 36/14 and reference 93-11	963	1,037	960	907
Coding region Variants	56,681	90,023	61,970	46,526
Homozygous variants	186,608	370,392	226,235	155,792
Heterozygous variants	167,732	183,071	170,743	137,445
Ti/Tv ratio	2.45	2.33	2.38	2.30
**InDel variants**				
Unique InDels	32,884	65,571	41,295	32,394
Unique InDels compared to JBT 36/14 and reference 93-11	100	162	115	102
Coding region Variants	6,531	12,300	7,540	6,183
Homozygous variants	24,953	54,979	32,638	24,983
Heterozygous variants	7,931	10,594	8,657	7,411

Intra-chromosomal and inter-chromosomal translocations were also observed in these mutant lines when compared to JBT 36/14 genome (Supplementary File 2). The number of inter-chromosomal translocations for lines 8, 9, 11, and 15 (i.e., 2,805, 2,816, 2,728, and 2,553, respectively) were higher than intra-chromosomal translocations in the lines (i.e., 2,200, 2,202, 2,169, and 2,116, respectively). The largest intra-chromosomal translocation was observed in lines 8, 9, and 11 as 45 Mb, whereas the largest intra-chromosomal translocation in line 15 was 31 Mb (Table 5). Among the mutant lines, ~72% of the SNP variations were present in the intergenic region (Figure 4A). In all lines, SNP variations in the introns (~15%) were higher than the exons (~10%). Splice site acceptors were affected by 210–365 SNPs in the lines. Up to ~4% of the SNP, variations were present in the 3′ UTR region in all lines, whereas ~2.5% of SNPs affected all lines’ 5′ UTR region (Figure 4A). Similarly, InDel variations in the intron (~16%) were higher than the exon (~11%) in all the lines. Splice site acceptors were affected by 25–57 variations in the lines. Up to 4% of the total InDel variations were present in the 3′ UTR region in all lines, whereas ~2% of the InDels affected all lines’ 5’ UTR region (Figure 4A).

**Table 5:** Structural variations in genomes of mutant line as compared to JBT 36/14.

Type of variation	Line-8	Line-9	Line-11	Line-15
Intra-chromosomal translocation	2,200	2,202	2,169	2,116
Inter-chromosomal translocation	2,805	2,816	2,728	2,553
Insertion	1	-	1	3
Deletion	5,271	5,292	5,260	5,083
Inversion	951	945	916	892

**Figure 5:**
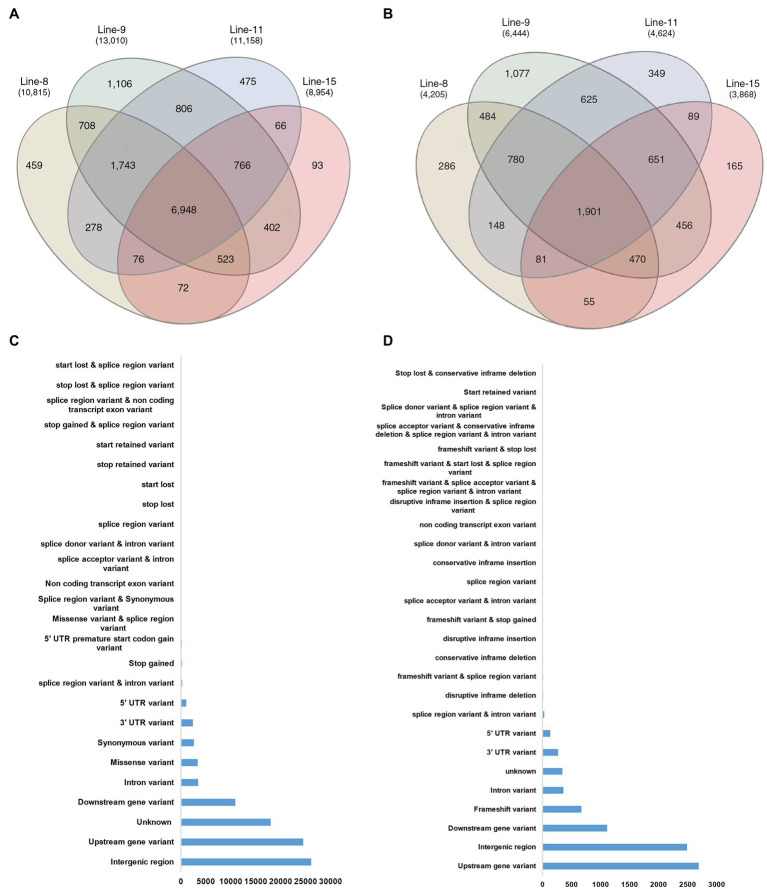
Common genes affected by SNPs and InDels in all mutant lines; **(A)** Venn diagram depicting common genes affected by SNP variants in mutant lines; **(B)** Common genes affected by InDel variants in mutant lines; **(C)** Annotation by location of common SNP variants at common genomic locations in all mutant lines; and **(D)** Annotation by location of common InDel variants at common genomic locations in all mutant lines.

The comparison of variants in the four lines showed that 93,224 SNPs and 8,170 InDels were found at common positions in all the lines. After that, the effects of variants on protein function were predicted and divided into four types (high, moderate, low, and modifier) based on the predicted severity of each effect (Supplementary File 3). Most variants belonged to the modifier category (70,609), such as intergenic region variants (26,088), upstream (24,490) and downstream gene variants (10,883), 3′ UTR (2,390), 5’ UTR ′(1,075) and intron variants (3,445). Variants that were inferred to have a low or weak impact (3,100 in number) mainly comprised synonymous variants (2,587), splice region variants, intron variants (243) and 5′ UTR premature start codon gain variants (167). The moderate effect group contained 3,410 SNP variants comprising mainly of missense variants (3,352). Notably, 316 SNP variants were predicted to have a high impact on protein function, the majority of which were 202 stop gained variants, which result in premature stop codons leading to disrupted transcription of genes. Among the 8,170 InDels found at a common position in all the lines, the majority belonged to the modifier category (7,397), which were identified upstream (2,690), downstream (1,112), intergenic regions (2,488), intron variants (359) and UTR variants (401). InDels with low and moderate effects were less in numbers, being 35 (InDels in splice region) and 32 variants (conservative and disruptive InDels). InDels causing frameshifts (671) were most frequent among those classified to have a high effect on protein function (706; Figures 5C,D; Supplementary File 3).

### Functional Annotation and Classification of Variations Common Between Mutant Lines

GO annotation, KEGG pathway, RiceCyc and Q-TARO analyses were carried out to annotate the genes affected with variations. Between the mutant lines, 6,948 genes contained SNPs, whereas 1,901 genes contained InDels (Figures 5A,B). Clubbed together, 7,331 genes common to all mutants showed SNPs or InDels compared to JBT 36/14, out of which 4,974 genes contained either SNPs or InDels at the same genomic location in all the mutants.

**Figure 6:**
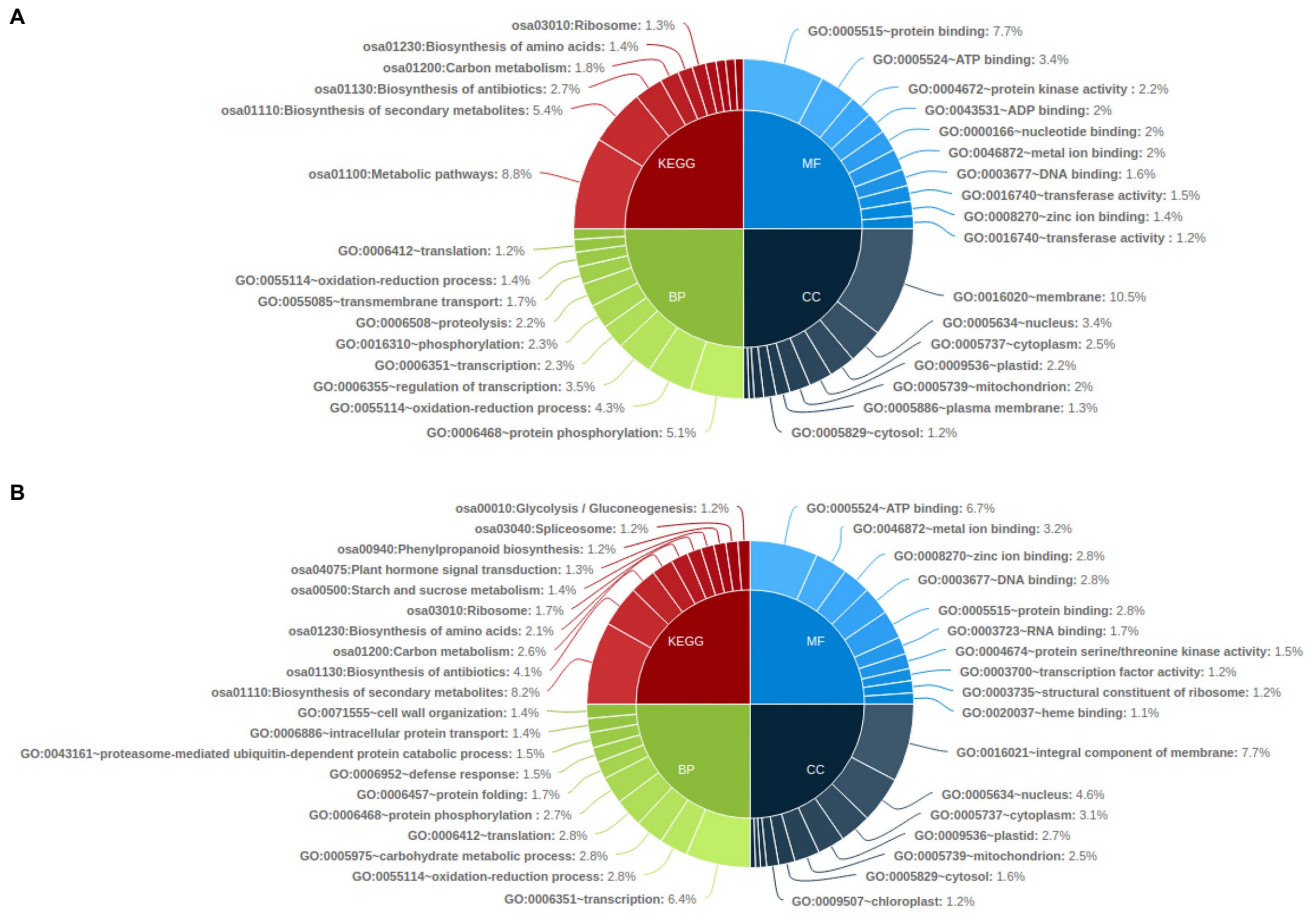
Functional annotation of common genes affected by genetic variations in all mutant lines by KEGG pathway and Gene Ontology analysis (top 10); **(A)** common genes affected by SNP in all mutant lines and **(B)** Common genes affected by InDels in all mutant lines.

KEGG pathway analysis of the common genes affected with genetic variations showed that metabolic pathways (osa01100) were the most affected with SNP affected genes followed by biosynthesis of secondary metabolites (osa01110) and biosynthesis of antibiotics pathway (osa01130). Biosynthesis of secondary metabolites (osa01110) was also highly enriched in InDel affected genes in all mutants, followed by biosynthesis of antibiotics (osa01130) and carbon metabolism pathway genes (osa01200; Figure 6A). Gene ontology enrichment analysis showed that SNP-affected genes were highly enriched in the biological process such as protein phosphorylation (GO:0006468), oxidation–reduction process (GO:0055114) and regulation of transcription related genes (GO:0006355). Among genes related to molecular function and cellular components, protein binding (GO:0005515) and membrane related (GO:0016020) genes were highly enriched. Meanwhile, InDel affected genes involved in biological processes were highly enriched in transcription (GO:0006351), oxidation–reduction process (GO:0055114) and carbohydrate metabolic process (GO:0005975). Among InDel containing genes related to molecular function and cellular components, ATP binding (GO:0005524) and Integral component of membrane (GO:0016021) related genes were highly enriched (Figure 6B).

**Figure 7:**
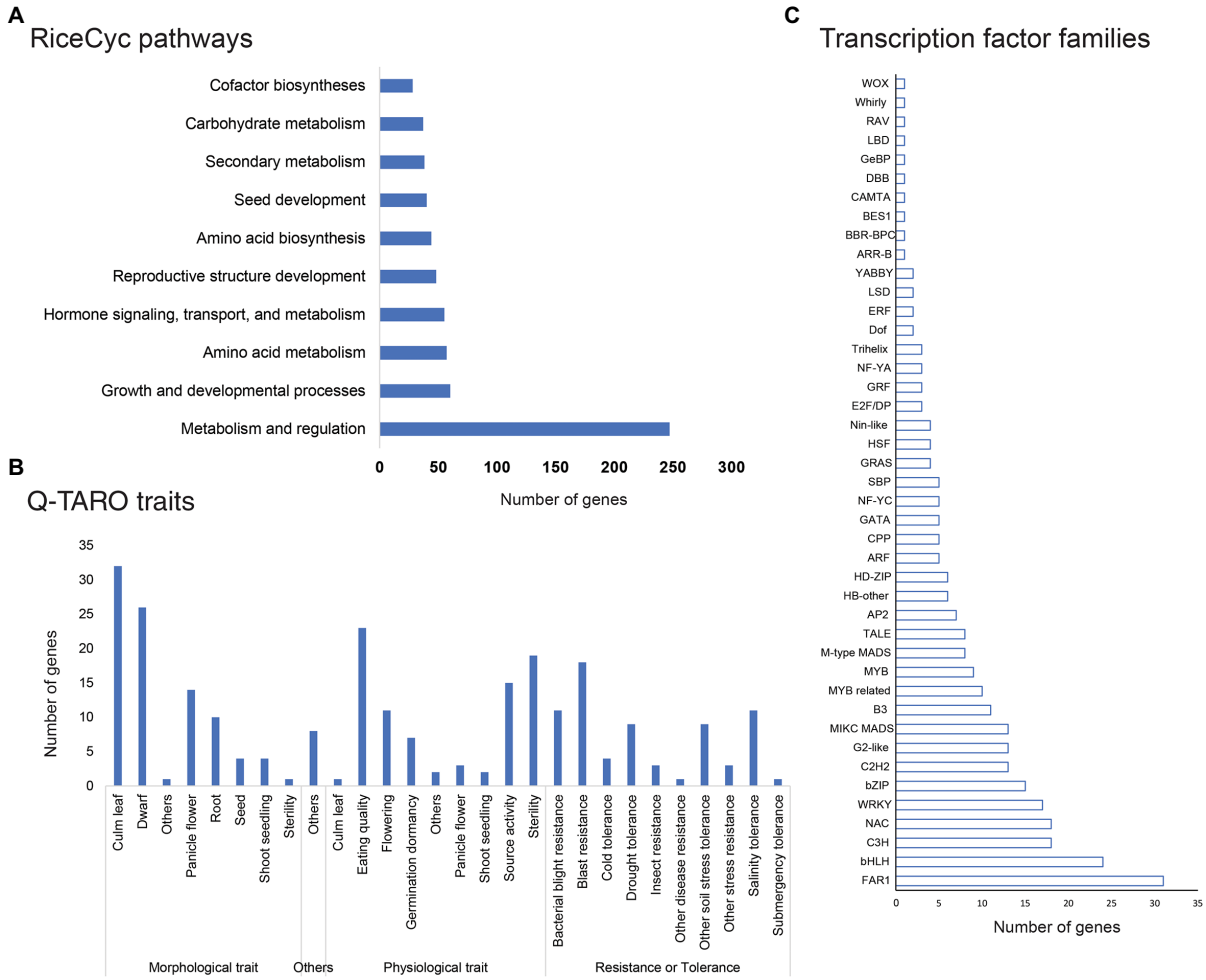
Pathway, trait and regulatory gene families affected by SNP or InDels; **(A)** Pathway analysis of common genes affected by genetic variations in all mutant lines; **(B)** common genes affected by genetic variations involved in major traits in rice; and **(C)** Common genes affected by SNP or InDel variations belonging to major rice transcription factor families (taken from PlantTFDB).

Pathway analysis of genes affected by genetic variations through RiceCyc led to mapping 1,350 genes on 212 pathways. The pathways with the highest number of variants affected genes included metabolism and regulation pathway (R-OSA-2744345, 247 genes), growth and developmental processes (R-OSA-9030769, 60 genes), amino acid metabolism (R-OSA-2744343, 57 genes), reproductive structure development (R-OSA-9031669) along with hormone signalling, transport, and metabolism (R-OSA-2744341; Figure 7A). Trait analysis using Q-TARO database mapped variant containing genes into four subgroups - morphological, physiological, resistance, or tolerance related genes and others. Q-TARO annotated a total of 253 genes, and the majority of them (92 genes) were grouped into morphological traits. Of these 92 genes, 32 were related to culm leaf, 24 to dwarf character governing genes, and 14 to panicle flowering. Second to morphological traits, 83 genes related to physiological traits such as eating quality (23), sterility (19) and source activity (15) were also affected. A total of 70 genes involved in several disease resistance or tolerance were identified, of which 18 genes were related to blast resistance, 15 to bacterial blight resistance and 11 genes to salinity tolerance (Figure 7B).

Many genes affected by variations belonged to several classes of transcription factors like FAR1, bHLH, NAC, bZIP, C3H, MIKC MADS, G2-like, WRKY, B3, C2H2, HB-other, M-type MADS, and MYB related transcription factors (Figure 7C). The highest number of variants containing genes were localized in the FAR-RED IMPAIRED RESPONSE 1 (FAR1) family of transcription factors (31), the bHLH class of transcription factors (24 genes) and NAC and C3H transcription factor family (18 genes each; Supplementary File 4).

Biotrophic pathogens typically interact with susceptibility genes (S) in the hosts to facilitate infection and disease development for a compatible interaction. Previously characterized S genes of *Arabidopsis thaliana* and *O. sativa* (van Schie and Takken, 2014) were used to find the homolog of those S genes in the *O. sativa* ssp. indica genome. We found 50 putative S genes with SNP variations and 31 with InDel variations. Among these, 23 S genes were affected by variants at common positions in all mutant lines with 53 SNPs and five InDels. Most of these variants were present in intergenic and 3′ UTR regions and were predicted to have modifier and moderate effects. *Arabidopsis* S genes like PME3, IOS1, PSY1R, CPR22, DMR6, FER, TOM1, RIN4, SSI1, FDH, CSLA9, AtNUDT6, SRFR1, TOM2A, PMR6, AtUBP13, RST1, PMR4, SIZ1, and CPR1 had modifier SNPs and InDels, whereas FAD8 had SNPs with predicted moderate effect (Supplementary File 4).

## Discussion

JBT 36/14 is an indica rice landrace that has been previously studied for its suitability for a promising trait donor for rice improvement programs (Shet et al., 2012; Mallikarjuna, 2013). JBT 36/14 showed tolerance to abiotic stress conditions (Raju et al., 2014; Basavaraju et al., 2020; Sampangi-Ramaiah et al., 2020) and brown planthopper (*Nilaparvata lugens L*.) resistance in previous studies (Dharshini and Gowda, 2014; Dharshini and Sidde, 2015; Raju et al., 2017). We sequenced the whole genome of a rice landrace JBT 36/14, and its activation tagged RRKN resistant mutant lines 8, 9, 11 and 15. We found the highest genetic variations in chr 1 in the parent JBT 36/14, as well as the mutants. The whole-genome sequencing of JBT 36/14 yielded 81.57 million paired-end reads of 151 bp, and 98.3% of HQ reads mapped to the reference indica genome. The SNP variants were mostly transitions, whereas most InDels were short, of 1–2 bp size. Nucleotide substitution analysis (the ratio of transitions to transversions (Ti / Tv)) was performed and found to be ~2.40. The mapped genome of JBT 36/14 revealed a clear bias toward transitions (more than twice that of transversions), and deviated from the expected ratio of 0.5 (Stoltzfus and McCandlish, 2017). This phenomenon is known as “transition bias,” which has previously been reported in rice (Morton, 1995). Higher Ti/Tv ratios have been previously reported in maize, oats, medicago, diploid wheat, *Triticum monococcum* and barley (Batley et al., 2003; Vitte and Bennetzen, 2006; Bindusree et al., 2017). The Ti/Tv ratio observed here is higher than some previous rice studies (Subbaiyan et al., 2012; Jain et al., 2014; Chai et al., 2018), suggesting a low level of genetic divergence in this landrace. Due to the wobble effect, transitions manifest primarily into silent mutations that do not alter the amino acid and thus conserve the amino acid chain (Wakeley, 1996). Moreover, Transitions are found to be more conservative than transversions (Stoltzfus and Norris, 2016). SNP and InDel variations in JBT 36/14 were observed to affect metabolic pathways and biosynthesis of metabolic pathways, affecting the increased production of phenolic compounds and reduced sugar content observed in the previous studies (Dharshini and Gowda, 2014). The draft genome sequence of JBT 36/14 is a good resource for understanding genotypic and phenotypic variations in rice and will enable its use in rice breeding programs (Shet et al., 2012).

Activation tagging is a robust forward genetics approach to generate genetic resistance against biotic and abiotic stresses and improve plant traits (Gao et al., 2014; Moin et al., 2016; Manimaran et al., 2017). Such mutants can help study traits that are hard to find in natural sources, for example, nematode resistance. Four activation tagged mutant lines showing RRKN resistance were identified and T-DNA insertion was confirmed in a previous study (Hatzade et al., 2019a). Here, we sequenced the genomes of these resistant mutant lines 8, 9, 11, and 15 to understand the genomic variations that might be responsible for imparting nematode resistance in addition to the T-DNA.

T-DNA insertions have been observed to cause chromosomal translocations and InDels in the target genome (Lafleuriel et al., 2004; Curtis et al., 2009; Ruprecht et al., 2014; Pucker et al., 2021). The whole-genome sequencing of mutant lines showed a high degree of genetic variations in terms of SNPs and InDels compared to its JBT 36/14 parent. The difference in number of variants in the mutants was directly proportional to the number of sequenced reads. The Ti/Tv ratio of >2 for all mutant lines was similar to JBT 36/14. However, unlike T → C transitions in JBT 36/14, G → A and C → T transition were the major types of transitions in mutants. Also, the highest number of variants was observed to be spanning chr number 1 and lowest in chr number 9. The increased number of small mutations and chromosomal translocations in these lines are unprecedented for T-DNA mutants as these are usually low in such mutants (Ossowski et al., 2010; Schouten et al., 2017).

Analysis of common genes containing variants in all the mutant lines suggested that the majority of the genes were involved in the metabolic pathway and gene regulation in rice. In particular, genes involved in the biosynthesis of secondary metabolites seemed to contain majority of common InDels and SNPs. This is interesting as a previous study regarding changes in transcriptomic profile in line-9 after nematode infection showed up-regulation of genes involved in the production of rice phytoalexins such as oryzalexins phytocassanes, momilactones and several flavonoid compounds (Dash et al., 2021). It may be suggested that in addition to the effect of activation tagging, these genomic structural variants may also be contributing to altered phytoalexin and flavonoid production in the mutants.

Several phenotypic variations were also observed in mutant lines compared to their JBT 36/14 wild type in green house conditions. These mutants varied compared to their wild type in terms of plant height, internodal length and width of leaf blade, number of tillers, flowering time, root structure and distinct seed color (Hatzade et al., 2019b). The morphological and physiological trait governing genes with the common variants might be associated with these different phenotypes observed in the mutants.

Common variants were also observed in several transcription factors (TF) families like FAR1, BHLH, and NAC in the mutants. FAR-RED IMPAIRED RESPONSE 1 (FAR1) transcription factor families play multiple roles in a wide range of cellular processes, including light signal transduction (Wang and Deng, 2002), circadian clock and flowering time regulation (Li et al., 2011), oxidative stress responses (Ma et al., 2016), and plant immunity (Wang et al., 2016). Similarly, rice BHLH transcription factors also have a role in both abiotic (Wang et al., 2003; Zhou et al., 2009; Sun et al., 2018) and biotic (Yamamura et al., 2015; Wei and Chen, 2018) stress responses of plants. NAC transcription factor is also known to be involved in abiotic and biotic stress responses in rice (Kaneda et al., 2009; Puranik et al., 2012). Some other TF families like WRKY, bzip, GATA, and MYB, which are related to plant stress responses, were also affected by variants in mutant plants. The majority of variants affecting TFs in mutants were predicted to be modifiers, while some had moderate effects. It may be suggested that some of these variations might be contributing to nematode stress response in the mutant lines in yet unknown ways.

Susceptibility genes play an important role in plant-pathogen interactions. All plant genes that facilitate infection and support compatibility can be considered as susceptibility (S) genes (Eckardt, 2002; van Schie and Takken, 2014). Mutation or loss of an S gene can limit the ability of the pathogen to cause disease, either due to impaired pre-penetration requirements such as host recognition and penetration or impaired post-penetration requirements like nutrients. Rice homologs of several *Arabidopsis* S genes were found to have common variants with predicted modifier and moderate effect in mutant lines. Among these S genes, PME3 is an S gene characterized in *Arabidopsis* targeted by Cyst nematode (*Heterodera schachtii*) cellulose-binding protein (Hewezi et al., 2008). Likewise, TOM1, TOM2A, and EIF4E are S genes targeted by viruses, while the rest of the identified S genes are targeted by either bacterial or fungal pathogens (van Schie and Takken, 2014). Nematodes secrete several effectors to establish feeding sites and facilitate penetration (Truong et al., 2015; Mejias et al., 2019). Effectors generally target S genes to facilitate disease progression. Variants in S genes can lead to a loss in its activity and may impede interaction with the pathogen—RRKN in this case. However, further validation is required to affirm the role of identified S genes in nematode resistance.

In summary, this study investigated genomic structural variations in rice landrace JBT 36/14 and its nematode-resistant activation tagged mutants. The genome of the rice landrace JBT 36/14 will add to the available databases of rice genetic variations. A set of 7,331 common genes affected by structural variations were recognized in all nematode-resistant mutant lines. These genes included secondary metabolite biosynthesis pathway genes, several families of TFs, and S genes. Further validation of these variants might help link them to the resistant phenotype observed in these mutants and may be helpful in future breeding programs.

## Data Availability Statement

The original contributions presented in the study are publicly available. This data can be found here: National Center for Biotechnology Information (NCBI) BioProject database under accession number PRJNA721239.

## Author Contributions

MD and UR conceived and designed the experiments and received the funding. MD performed the experiments. MD, VS, RB, and JG analysed the data. MD, VS, and UR wrote the manuscript. MD, VS, RS, and UR revised the manuscript. All authors contributed to the article and approved the submitted version.

## Funding

The work was funded by the NAHEP-CAAST project on Genomics-Assisted Crop Improvement and Management grant to MD. This research was also supported by the Department of Biotechnology, Ministry of Science and Technology, India grant no. BT/PR18924/COE/34/48/2017 to UR.

## Conflict of Interest

JG and RB were employed by Bionivid Technology Private Limited.

The remaining authors declare that the research was conducted in the absence of any commercial or financial relationships that could be construed as a potential conflict of interest.

## Publisher’s Note

All claims expressed in this article are solely those of the authors and do not necessarily represent those of their affiliated organizations, or those of the publisher, the editors and the reviewers. Any product that may be evaluated in this article, or claim that may be made by its manufacturer, is not guaranteed or endorsed by the publisher.

## Footnotes

^1^ plants.ensembl.org/Oryza_indica/Tools/VEP

## Supplementary Material

Supplementary File 1 SNP and InDel variants identified in JBT 36/14 as compared to the *O. sativa* ssp. indica reference 93-11.

Supplementary File 2 Structural variations identified in mutant lines as compared to JBT 36/14.

Supplementary File 3 SNP and InDel variants found at common genomic locations in all mutant lines.

Supplementary File 4 Variant containing putative susceptibility genes and transcription factors families identified in all mutant lines.

## References

[ref1] An G. Lee S. Kim S.-H. Kim S.-R. (2005). Molecular genetics using T-DNA in Rice. Plant Cell Physiol. 46, 14–22. doi: 10.1093/pcp/pci502 15659434

[ref2] Basavaraju S. N. Lakshmikanth R. Y. Kumaraswamy R. Makarla U. (2020). Genotypes with enhanced expressions of acquired tolerance mechanisms showed improved growth under stress. Plant Physiol. Rep. 25, 9–23. doi: 10.1007/s40502-019-00482-8

[ref3] Batley J. Barker G. O’Sullivan H. Edwards K. J. Edwards D. (2003). Mining for single nucleotide polymorphisms and insertions/deletions in maize expressed sequence tag data. Plant Physiol. 132, 84–91. doi: 10.1104/pp.102.019422, PMID: 12746514 PMC166954

[ref4] Bindusree G. Natarajan P. Kalva S. Madasamy P. (2017). Whole genome sequencing of *Oryza sativa* L. cv. Seeragasamba identifies a new fragrance allele in rice. PLoS One 12:e0188920. doi: 10.1371/journal.pone.0188920, PMID: 29190814 PMC5708779

[ref5] Cabasan M. T. N. Kumar A. De Waele D. (2018). Evaluation of resistance and tolerance of rice genotypes from crosses of *Oryza glaberrima* and *O. sativa* to the rice root-knot nematode, *Meloidogyne graminicola*. Trop. Plant Pathol. 43, 230–241. doi: 10.1007/s40858-018-0210-8

[ref6] Chai C. Shankar R. Jain M. Subudhi P. K. (2018). Genome-wide discovery of DNA polymorphisms by whole genome sequencing differentiates weedy and cultivated rice. Sci. Rep. 8:14218. doi: 10.1038/s41598-018-32513-z, PMID: 30242197 PMC6155081

[ref7] Cingolani P. Platts A. Wang L. L. Coon M. Nguyen T. Wang L.. (2012). A program for annotating and predicting the effects of single nucleotide polymorphisms. SnpEff. Fly 6, 80–92. doi: 10.4161/fly.19695, PMID: 22728672 PMC3679285

[ref8] Curtis M. J. Belcram K. Bollmann S. R. Tominey C. M. Hoffman P. D. Mercier R.. (2009). Reciprocal chromosome translocation associated with TDNA-insertion mutation in Arabidopsis: genetic and cytological analyses of consequences for gametophyte development and for construction of doubly mutant lines. Planta 229, 731–745. doi: 10.1007/s00425-008-0868-0, PMID: 19082841 PMC4186712

[ref9] Dash M. Somvanshi V. S. Budhwar R. Godwin J. Shukla R. N. Rao U. (2021). A rice root-knot nematode *Meloidogyne graminicola*-resistant mutant rice line shows early expression of plant-defence genes. Planta 253:108. doi: 10.1007/s00425-021-03625-0, PMID: 33866432

[ref10] Dharmawardhana P. Ren L. Amarasinghe V. Monaco M. Thomason J. Ravenscroft D.. (2013). A genome scale metabolic network for rice and accompanying analysis of tryptophan, auxin and serotonin biosynthesis regulation under biotic stress. Rice 6:15. doi: 10.1186/1939-8433-6-15, PMID: 24280345 PMC4883713

[ref11] Dharshini G. M. Gowda D. K. S. (2014). Biochemical basis of resistance in rice landraces to brown planthopper, *Nilaparvata lugens* (Stal.). Curr. Biot. 8, 213–219.

[ref12] Dharshini G. M. Sidde G. D. K. (2015). Reaction of rice landraces to the brown plant hopper, *Nilaparvata lugens* (Homoptera: Delphacidae). Trends Biosci. 8, 4164–4168.

[ref13] Eckardt N. A. (2002). Plant disease susceptibility genes? Plant Cell 14, 1983–1986. doi: 10.1105/tpc.140910, PMID: 12215498 PMC543214

[ref14] Galeng-Lawilao J. Kumar A. De Waele D. (2018). QTL mapping for resistance to and tolerance for the rice root-knot nematode, *Meloidogyne graminicola*. BMC Genet. 19:53. doi: 10.1186/s12863-018-0656-1, PMID: 30081817 PMC6080554

[ref15] Gao D. Appiano M. Huibers R. P. Chen X. Loonen A. E. H. M. Visser R. G. F.. (2014). Activation tagging of ATHB13 in *Arabidopsis thaliana* confers broad-spectrum disease resistance. Plant Mol. Biol. 86, 641–653. doi: 10.1007/s11103-014-0253-2, PMID: 25293871

[ref16] Haraksingh R. R. Snyder M. P. (2013). Impacts of variation in the human genome on gene regulation. J. Mol. Biol. 425, 3970–3977. doi: 10.1016/j.jmb.2013.07.015 23871684

[ref17] Hatzade B. Singh D. Phani V. Kumbhar S. Rao U. (2020). Profiling of defense responsive pathway regulatory genes in Asian rice (*Oryza sativa*) against infection of *Meloidogyne graminicola* (Nematoda:Meloidogynidae). 3. Biotech 10:60. doi: 10.1007/s13205-020-2055-3, PMID: 32030329 PMC6977811

[ref18] Hatzade B. Sreevathsa R. Makarla U. Rao U. (2019a). T-DNA activation tagging in rice results in a variable response to *Meloidogyne graminicola* infection. Biologia 74, 1197–1217. doi: 10.2478/s11756-019-00281-4

[ref19] Hatzade B. Sreevathsa R. Makarla U. Rao U. (2019b). Evaluation of activation tagged rice mutants for variability in response to *Meloidogyne graminicola* under challenged inoculation. Indian. J. Genet. Plant. Breed. 79, 685–692. doi: 10.31742/IJGPB.79.4.6

[ref20] Hayashi K. Yoshida H. Ashikawa I. (2006). Development of PCR-based allele-specific and InDel marker sets for nine rice blast resistance genes. Theor. Appl. Genet. 113, 251–260. doi: 10.1007/s00122-006-0290-6, PMID: 16791691

[ref21] Hewezi T. Howe P. Maier T. R. Hussey R. S. Mitchum M. G. Davis. (2008). Cellulose binding protein from the parasitic nematode *Heterodera schachtii* interacts with Arabidopsis pectin methylesterase: cooperative cell wall modification during parasitism. Plant Cell 20, 3080–3093. doi: 10.1105/tpc.108.063065, PMID: 19001564 PMC2613657

[ref22] Howe K. L. Contreras-Moreira B. De Silva N. Maslen G. Akanni W. Allen J.. (2020). Ensembl genomes 2020—enabling non-vertebrate genomic research. Nucleic Acids Res. 48, D689–D695. doi: 10.1093/nar/gkz890, PMID: 31598706 PMC6943047

[ref23] Huang X. Lu T. Han B. (2013). Resequencing rice genomes: An emerging new era of rice genomics. Trends Genet. 29, 225–232. doi: 10.1016/j.tig.2012.12.001, PMID: 23295340

[ref24] Huang D. W. Sherman B. T. Lempicki R. A. (2009). Systematic and integrative analysis of large gene lists using DAVID bioinformatics resources. Nat. Protoc. 4, 44–57. doi: 10.1038/nprot.2008.211, PMID: 19131956

[ref25] Jain M. Moharana K. C. Shankar R. Kumari R. Garg R. (2014). Genome-wide discovery of DNA polymorphisms in rice cultivars with contrasting drought and salinity stress response and their functional relevance. Plant Biotechnol. J. 12, 253–264. doi: 10.1111/pbi.12133, PMID: 24460890

[ref26] Jin J. Tian F. Yang D.-C. Meng Y.-Q. Kong L. Luo J.. (2017). PlantTFDB 4.0: Toward a central hub for transcription factors and regulatory interactions in plants. Nucleic Acids Res. 45, D1040–D1045. doi: 10.1093/nar/gkw982, PMID: 27924042 PMC5210657

[ref01] Jones N. Ougham H. Thomas H. Pašakinskienė I. (2009). Markers and mapping revisited: finding your gene. New Phytol. 183, 935–966. doi: 10.1111/j.1469-8137.2009.02933.x, PMID: 19594696

[ref27] Kaneda T. Taga Y. Takai R. Iwano M. Matsui H. Takayama S.. (2009). The transcription factor OsNAC4 is a key positive regulator of plant hypersensitive cell death. EMBO J. 28, 926–936. doi: 10.1038/emboj.2009.39, PMID: 19229294 PMC2670867

[ref28] Khush G. S. (2013). Strategies for increasing the yield potential of cereals: case of rice as an example. Plant Breed. 132, 433–436. doi: 10.1111/pbr.1991

[ref29] Koboldt D. C. Zhang Q. Larson D. E. Shen D. McLellan M. D. Lin L.. (2012). VarScan 2: somatic mutation and copy number alteration discovery in cancer by exome sequencing. Genome Res. 22, 568–576. doi: 10.1101/gr.129684.111, PMID: 22300766 PMC3290792

[ref30] Krzywinski M. Schein J. Birol İ. Connors J. Gascoyne R. Horsman D.. (2009). Circos: An information aesthetic for comparative genomics. Genome Res. 19, 1639–1645. doi: 10.1101/gr.092759.109, PMID: 19541911 PMC2752132

[ref31] Kumar V. Khan M. R. Walia R. K. (2020). Crop loss estimations due to plant-parasitic nematodes in major crops in India. Natl. Acad. Sci. Lett. 43, 409–412. doi: 10.1007/s40009-020-00895-2

[ref32] Kyndt T. Fernandez D. Gheysen G. (2014). Plant-parasitic nematode infections in Rice: molecular and cellular insights. Annu. Rev. Phytopathol. 52, 135–153. doi: 10.1146/annurev-phyto-102313-050111, PMID: 24906129

[ref33] Lafleuriel J. Degroote F. Depeiges A. Picard G. (2004). A reciprocal translocation, induced by a canonical integration of a single T-DNA, interrupts the HMG-I/Y Arabidopsis thaliana gene. Plant Physiol. Biochem. 42, 171–179. doi: 10.1016/j.plaphy.2004.01.003, PMID: 15051040

[ref34] Li H. (2013). Aligning sequence reads, clone sequences and assembly contigs with BWA-MEM. arXiv [Preprint].

[ref35] Li H. Handsaker B. Wysoker A. Fennell T. Ruan J. Homer N.. (2009). The sequence alignment/map format and SAMtools. Bioinformatics 25, 2078–2079. doi: 10.1093/bioinformatics/btp352, PMID: 19505943 PMC2723002

[ref36] Li G. Siddiqui H. Teng Y. Lin R. Wan X. Li J.. (2011). Coordinated transcriptional regulation underlying the circadian clock in Arabidopsis. Nat. Cell Biol. 13, 616–622. doi: 10.1038/ncb2219, PMID: 21499259

[ref37] Liang F. Xin X. Hu Z. Xu J. Wei G. Qian X.. (2011). Genetic analysis and fine mapping of a novel Semidominant dwarfing gene LB4D in Rice. J. Integr. Plant Biol. 53, 312–323. doi: 10.1111/j.1744-7909.2011.01031.x, PMID: 21294842

[ref38] Lin M. Whitmire S. Chen J. Farrel A. Shi X. Guo J. (2017). Effects of short indels on protein structure and function in human genomes. Sci. Rep. 7:9313. doi: 10.1038/s41598-017-09287-x, PMID: 28839204 PMC5570956

[ref39] Luc M. Sikora R. A. Bridge J. (Eds.) (2005). Plant Parasitic Nematodes in Subtropical and Tropical Agriculture. 2nd Edn. Wallingford: CABI. doi: 10.1079/9780851997278.0000

[ref40] Ma L. Tian T. Lin R. Deng X.-W. Wang H. Li G. (2016). Arabidopsis FHY3 and FAR1 regulate light-induced myo -inositol biosynthesis and oxidative stress responses by transcriptional activation of MIPS1. Mol. Plant 9, 541–557. doi: 10.1016/j.molp.2015.12.013, PMID: 26714049

[ref41] Mallikarjuna N.M. (2013) Genetic analysis and breeding potential for grain yield and its stabilizing traits under aerobic condition in rice (*Oryza sativa* L.). Ph.D. thesis. University of Agricultural Sciences, Bangaluru, Karnataka.

[ref42] Manimaran P. Venkata Reddy S. Moin M. Raghurami Reddy M. Yugandhar P. Mohanraj S. S.. (2017). Activation-tagging in indica rice identifies a novel transcription factor subunit, NF-YC13 associated with salt tolerance. Sci. Rep. 7:9341. doi: 10.1038/s41598-017-10022-9, PMID: 28839256 PMC5570948

[ref43] Mantelin S. Bellafiore S. Kyndt T. (2017). *Meloidogyne graminicola*: A major threat to rice agriculture. Mol. Plant Pathol. 18, 3–15. doi: 10.1111/mpp.12394, PMID: 26950515 PMC6638252

[ref44] McCouch S. R. Zhao K. Wright M. Tung C.-W. Ebana K. Thomson M.. (2010). Development of genome-wide SNP assays for rice. Breed. Sci. 60, 524–535. doi: 10.1270/jsbbs.60.524

[ref45] Mejias J. Truong N. M. Abad P. Favery B. Quentin M. (2019). Plant proteins and processes targeted by parasitic nematode effectors. Front. Plant Sci. 10:970. doi: 10.3389/fpls.2019.00970, PMID: 31417587 PMC6682612

[ref46] Moin M. Bakshi A. Saha A. Udaya Kumar M. Reddy A. R. Rao K. V.. (2016). Activation tagging in indica rice identifies ribosomal proteins as potential targets for manipulation of water-use efficiency and abiotic stress tolerance in plants. Plant Cell Environ. 39, 2440–2459. doi: 10.1111/pce.12796, PMID: 27411514

[ref47] Morton B. R. (1995). Neighboring base composition and transversion/transition bias in a comparison of rice and maize chloroplast noncoding regions. Proc. Natl. Acad. Sci. 92, 9717–9721. doi: 10.1073/pnas.92.21.9717, PMID: 7568204 PMC40873

[ref48] Ossowski S. Schneeberger K. Lucas-Lledó J. I. Warthmann N. Clark R. M. Shaw R. G.. (2010). The rate and molecular spectrum of spontaneous mutations in *Arabidopsis thaliana*. Science 327, 92–94. doi: 10.1126/science.1180677, PMID: 20044577 PMC3878865

[ref49] Pan C. Li A. Dai Z. Zhang H. Liu G. Wang Z.. (2008). InDel and SNP markers and their applications in map-based cloning of Rice genes. Rice Sci. 15, 251–258. doi: 10.1016/S1672-6308(09)60001-9

[ref50] Patel R. K. Jain M. (2012). NGS QC toolkit: A toolkit for quality control of next generation sequencing data. PLoS One 7:e30619. doi: 10.1371/journal.pone.0030619, PMID: 22312429 PMC3270013

[ref51] Plowright R. A. Coyne D. L. Nash P. Jones M. P. (1999). Resistance to the rice nematodes *Heterodera sacchari*, *Meloidogyne graminicola* and *M. incognita* in *Oryza glaberrima* and *O. glaberrima* x *O. sativa* interspecific hybrids. Nematology 1, 745–751. doi: 10.1163/156854199508775

[ref52] Pucker B. Kleinbölting N. Weisshaar B. (2021). Large scale genomic rearrangements in selected *Arabidopsis thaliana* T-DNA lines are caused by T-DNA insertion mutagenesis. BMC Genomics 22:599. doi: 10.1186/s12864-021-07877-8, PMID: 34362298 PMC8348815

[ref53] Puranik S. Sahu P. P. Srivastava P. S. Prasad M. (2012). NAC proteins: regulation and role in stress tolerance. Trends Plant Sci. 17, 369–381. doi: 10.1016/j.tplants.2012.02.004 22445067

[ref54] Raju B. R. Narayanaswamy B. R. Mohankumar M. V. Sumanth K. K. Rajanna M. P. Mohanraju B.. (2014). Root traits and cellular level tolerance hold the key in maintaining higher spikelet fertility of rice under water limited conditions. Funct. Plant Biol. 41, 930–939. doi: 10.1071/FP13291, PMID: 32481046

[ref55] Raju P. G. Shivashankar T. Chandappa L. (2017). Genetic basis of resistance to Brown Plant hopper (*Nilaparvata lugens* Stal) in local landraces of Rice. Int. J. Curr. Microbiol. Appl. Sci. 6, 3388–3393. doi: 10.20546/ijcmas.2017.608.405

[ref56] Ruden D. Cingolani P. Patel V. Coon M. Nguyen T. Land S.. (2012). Using *Drosophila melanogaster* as a model for Genotoxic chemical mutational studies with a new program, SnpSift. Front. Genet. 3:35. doi: 10.3389/fgene.2012.00035, PMID: 22435069 PMC3304048

[ref57] Ruprecht C. Carroll A. Persson S. (2014). T-DNA-induced chromosomal translocations in feronia and anxur2 mutants reveal implications for the mechanism of collapsed pollen due to chromosomal rearrangements. Mol. Plant 7, 1591–1594. doi: 10.1093/mp/ssu062, PMID: 24874868

[ref58] Sampangi-Ramaiah M. H. Jagadheesh D. P. Jambagi S. Vasantha Kumari M. M. Oelmüller R. Nataraja K. N.. (2020). An endophyte from salt-adapted Pokkali rice confers salt-tolerance to a salt-sensitive rice variety and targets a unique pattern of genes in its new host. Sci. Rep. 10:3237. doi: 10.1038/s41598-020-59998-x, PMID: 32094443 PMC7039991

[ref59] Schouten H. J. vande Geest H. Papadimitriou S. Bemer M. Schaart J. G. Smulders M. J. M.. (2017). Resequencing transgenic plants revealed rearrangements at T-DNA inserts, and integration of a short T-DNA fragment, but no increase of small mutations elsewhere. Plant Cell Rep. 36, 493–504. doi: 10.1007/s00299-017-2098-z, PMID: 28155116 PMC5316556

[ref60] Shastry B. S. (2009). “SNPs: impact on gene function and phenotype,” in Single Nucleotide Polymorphisms: Methods and Protocols. ed. Komar A. A. (Humana Press), 3–22. doi: 10.1007/978-1-60327-411-1_1 19768584

[ref61] Shet R. M. Rajanna M. P. Ramesh S. Sheshshayee M. S. Mahadevu P. (2012). Genetic variability, correlation and path coefficient studies in F2 generation of aerobic rice (*Orzya sativa* L.). Electron. J. Plant Breed. 3, 925–931.

[ref62] Steele K. A. Ogden R. McEwing R. Briggs H. Gorham J. (2008). InDel markers distinguish Basmatis from other fragrant rice varieties. Field Crops Res. 105, 81–87. doi: 10.1016/j.fcr.2007.08.001

[ref63] Stoltzfus A. McCandlish D. M. (2017). Mutational biases influence parallel adaptation. Mol. Biol. Evol. 34, 2163–2172. doi: 10.1093/molbev/msx180, PMID: 28645195 PMC5850294

[ref64] Stoltzfus A. Norris R. W. (2016). On the causes of evolutionary transition:Transversion bias. Mol. Biol. Evol. 33, 595–602. doi: 10.1093/molbev/msv274, PMID: 26609078 PMC7107541

[ref65] Subbaiyan G. K. Waters D. L. E. Katiyar S. K. Sadananda A. R. Vaddadi S. Henry R. J. (2012). Genome-wide DNA polymorphisms in elite indica rice inbreds discovered by whole-genome sequencing. Plant Biotechnol. J. 10, 623–634. doi: 10.1111/j.1467-7652.2011.00676.x, PMID: 22222031

[ref66] Sun X. Wang Y. Sui N. (2018). Transcriptional regulation of bHLH during plant response to stress. Biochem. Biophys. Res. Commun. 503, 397–401. doi: 10.1016/j.bbrc.2018.07.123 30057319

[ref67] Truong N. M. Nguyen C.-N. Abad P. Quentin M. Favery B. (2015). “Chapter twelve—function of root-knot nematode effectors and their targets in plant parasitism,” in Adv. Bot. Res. eds. Escobar C. Fenoll C., Vol. 73 (Academic Press), 293–324. doi: 10.1016/bs.abr.2014.12.010

[ref68] van Schie C. C. N. Takken F. L. W. (2014). Susceptibility genes 101: how to be a good host. Annu. Rev. Phytopathol. 52, 551–581. doi: 10.1146/annurev-phyto-102313-045854, PMID: 25001453

[ref69] Varshney R. K. Nayak S. N. May G. D. Jackson S. A. (2009). Next-generation sequencing technologies and their implications for crop genetics and breeding. Trends Biotechnol. 27, 522–530. doi: 10.1016/j.tibtech.2009.05.006, PMID: 19679362

[ref70] Vasemägi A. Gross R. Palm D. Paaver T. Primmer C. R. (2010). Discovery and application of insertion-deletion (INDEL) polymorphisms for QTL mapping of early life-history traits in Atlantic salmon. BMC Genomics 11:156. doi: 10.1186/1471-2164-11-156, PMID: 20210987 PMC2838853

[ref71] Vitte C. Bennetzen J. L. (2006). Analysis of retrotransposon structural diversity uncovers properties and propensities in angiosperm genome evolution. Proc. Natl. Acad. Sci. 103, 17638–17643. doi: 10.1073/pnas.0605618103, PMID: 17101966 PMC1693799

[ref72] Wakeley J. (1996). The excess of transitions among nucleotide substitutions: new methods of estimating transition bias underscore its significance. Trends Ecol. Evol. 11, 158–162. doi: 10.1016/0169-5347(96)10009-4, PMID: 21237791

[ref73] Wang H. Deng X. W. (2002). *Arabidopsis* FHY3 defines a key phytochrome A signaling component directly interacting with its homologous partner FAR1. EMBO J. 21, 1339–1349. doi: 10.1093/emboj/21.6.1339, PMID: 11889039 PMC125923

[ref74] Wang W. Tang W. Ma T. Niu D. Jin J. B. Wang H.. (2016). A pair of light signaling factors FHY3 and FAR1 regulates plant immunity by modulating chlorophyll biosynthesis. J. Integr. Plant Biol. 58, 91–103. doi: 10.1111/jipb.12369, PMID: 25989254 PMC4736690

[ref75] Wang Y.-J. Zhang Z.-G. He X.-J. Zhou H.-L. Wen Y.-X. Dai J.-X.. (2003). A rice transcription factor OsbHLH1 is involved in cold stress response. Theor. Appl. Genet. 107, 1402–1409. doi: 10.1007/s00122-003-1378-x, PMID: 12920519

[ref76] Wei K. Chen H. (2018). Comparative functional genomics analysis of bHLH gene family in rice, maize and wheat. BMC Plant Biol. 18:309. doi: 10.1186/s12870-018-1529-5, PMID: 30497403 PMC6267037

[ref77] Yamamura C. Mizutani E. Okada K. Nakagawa H. Fukushima S. Tanaka A.. (2015). Diterpenoid phytoalexin factor, a bHLH transcription factor, plays a central role in the biosynthesis of diterpenoid phytoalexins in rice. Plant J. 84, 1100–1113. doi: 10.1111/tpj.13065, PMID: 26506081

[ref78] Yonemaru J. Yamamoto T. Fukuoka S. Uga Y. Hori K. Yano M. (2010). Q-TARO: QTL annotation Rice online database. Rice 3, 194–203. doi: 10.1007/s12284-010-9041-z

[ref79] Zhan L. Ding Z. Peng D. Peng H. Kong L. Liu S.. (2018). Evaluation of Chinese rice varieties resistant to the root-knot nematode *Meloidogyne graminicola*. J. Integr. Agric. 17, 621–630. doi: 10.1016/S2095-3119(17)61802-1

[ref80] Zhou J. Li F. Wang J. Ma Y. Chong K. Xu Y. (2009). Basic helix-loop-helix transcription factor from wild rice (OrbHLH2) improves tolerance to salt- and osmotic stress in Arabidopsis. J. Plant Physiol. 166, 1296–1306. doi: 10.1016/j.jplph.2009.02.007, PMID: 19324458

